# Oxygen Vacancy Evolution at Li_*x*_V_2_O_5_/LiPON Solid State Electrochemical Interfaces Using Depth Resolved Cathodoluminescence Spectroscopy

**DOI:** 10.1021/acsami.5c25104

**Published:** 2026-04-17

**Authors:** Daniel Halbing, Gregory Pustorino, Leopoldo Tapia-Aracayo, David Stewart, Yue Qi, Leonard J. Brillson

**Affiliations:** † Department of Physics, 2647 The Ohio State University, Columbus, Ohio 43210, United States; ‡ Department of Engineering, 6752 Brown University, Providence, Rhode Island 02912, United States; § Department of Materials Science and Engineering and Institute for Research in Electronics and Applied Physics, 1068 University of Maryland, College Park, Maryland 20742, United States; ∥ Department of Electrical and Computer Engineering, 2647 The Ohio State University, Columbus, Ohio 43210, United States

**Keywords:** charged point defects, electrochemical interfaces, diffusion barriers, DRCLS, DFT, oxygen vacancy, V_2_O_5_, LiPON

## Abstract

The formation of oxygen vacancies at buried LiPON/Li_*x*_V_2_O_5_ interfaces has been observed on a near-nanometer scale and nondestructively using depth-resolved cathodoluminescence spectroscopy (DRCLS) and interfacial markers. Before electrochemical cycling, as-deposited LiPON/Li_*x*_V_2_O_5_ exhibits a 1.6 eV defect optical emission, which density functional theory calculations identify as originating from oxygen vacancies. This defect appears first within a few nanometers of the buried LiPON/Li_*x*_V_2_O_5_ interface without cycling, indicating that spontaneous O diffusion from the Li_*x*_V_2_O_5_ lattice into LiPON may have caused these interface-localized oxygen vacancy defects. DRCLS measured the intensity and spatial distribution of this oxygen vacancy signal as a function of electrochemical cycling in a LiPON/Li_*x*_V_2_O_5_ half-cell, showing oxygen vacancy signal increasing and moving deeper into the electrode with increased cycle number. Significant electrochemical irreversibility was also observed, with poor Coulombic efficiency and a 15% drop in capacity over 50 cycles. Theoretical simulations predict that the presence of oxygen vacancies increases the energy barrier for lithium diffusion significantly, indicating that this aggregation of oxygen vacancies could be another battery degradation mechanism accompanying lithiation induced phase changes.

## Graphical abstract

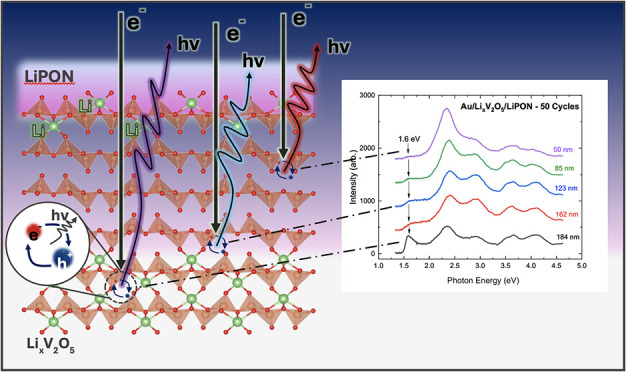

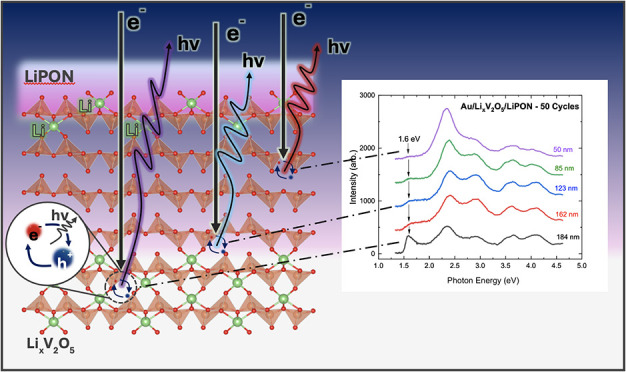

## Introduction

The nature of native point defect formation, growth, and movement at solid-state electrochemical interfaces has been a significant focus of solid-state electrochemical research, both at the fundamental and technological levels. The impact of electrically active defects with intertwined ion-electron behavior inside electrolyte and electrode materials can now be studied at the nanoscale level by advanced electron microscopy techniques combined with theoretical techniques and thin film model systems. Using depth-resolved cathodoluminescence spectroscopy (DRCLS), we report the first direct and noninvasive measurements of oxygen vacancy (V_O_) formation, accumulation and movement at the LiPON/Li_*x*_V_2_O_5_ (LVO) buried interface. These observations can strongly affect the movement of ions through electrolyte/electrode interfaces and their subsequent degradation with cycling.

Numerous electrochemical, physical, and materials science techniques have been used to probe solid-state electrochemical (SSEC) interfaces for the presence of electrically active defects, in particular, oxygen vacancies in metal oxides. Such oxygen vacancies are believed to be energetically favorable during Li-ion insertion into Li_*x*_V_2_O_5_ due to the reduction of V and due to Li–O bond formation, which allows for the displacement of the oxygen from their equilibrium metal-oxide lattice sites. This is different from layered transition metal oxides (e.g., LiCoO_2_), in which oxygen vacancies form at low Li content.^1^ Oxygen vacancies can also be generated due to heterogeneous interface formation, requiring in situ interface characterizations. Besides free surface V_2_O_5_ studies,^2^ most interface experimental techniques have been destructive, involving sputter profiling or cleaving to expose cross sections for X-ray photoelectron spectroscopy (XPS), secondary ion mass spectroscopy (SIMS), and photoluminescence spectroscopy (PLS).^2–4^ Cleavage damage, ion impact displacement, and air exposure can introduce additional electronic features and complexity. One significant nondestructive study of oxygen vacancies below the V_2_O_5_ surface has been a PL study of reduced V_2_O_5_, showing the appearance of 0.8 eV emission in V_2_O_5‑x_. compared with the 1.9–2.1 eV V_2_O_5_ band gap.^5^

Electronic structure studies of charge-neutral oxygen vacancies in V_2_O_5_ began before 1980.^6^ Density functional theory (DFT) calculations of reduced V_2_O_5_ showed that oxygen vacancy configurations can induce considerable local structural distortions,^7^ and affect Li-ion diffusion.^8^ Scanlon et al. demonstrated that DFT calculations with Hubbard-U corrections can capture the midgap states due to oxygen vacancies in V_2_O_5_.^9^ Further DFT+U studies have shown the formation of midbandgap states by oxygen vacancies in both γ-V_2_O_5_ and lithiated γ-LiV_2_O_5_.^10^ Work by Eyert and Höck^11^ provided density of states (DOS) calculations for bulk V_2_O_5_, which correlate closely with the DOS transition energies measured by DRCLS.^12^

In contrast to sputter profiling and cleavage, depth-resolved cathodoluminescence spectroscopy (DRCLS) with incident beam energies below hundred KeV is nondestructive, involving incident beam voltages, *E*_B_, ranging from below 100 eV to 25 keV, more than an order of magnitude below the MeV beam voltages which can cause atom displacement damage.^13^ Within this lower *E*_B_ range, DRCLS has been used to measure electronic and structural properties of lithiated LiPON/Li_*x*_V_2_O_5_ junctions buried below free surfaces.^12,14,15^

DRCLS employs an incident electron beam focused onto a sample, generating a cascade of secondary electrons through impact ionization. This process is shown schematically in Figure 1, with incident electron beams penetrating a Li_*x*_V_2_O_5_ sample and luminescence being produced from electron–hole pair recombination from defects and band-to-band transitions. Each feature and defect in a material has a characteristic luminescence energy which is read out in the form of an optical spectrum. The incident electron beam energy, *E*_B_, can be tuned from 0.5 to 5 keV in steps of as low as 0.1 keV to achieve near-nanometer depth-dependence,^16^ as higher incident beam energies probe deeper into the sample.^17,18^ Figure 1 shows an example of this depth dependence with two DRCLS spectra at different beam energies, one whose Bohr–Bethe range, *R*_B_, penetrates to the interface between LiPON/Li_*x*_V_2_O_5_ and one which penetrates deep into the bulk of the V_2_O_5_ layer. When increasing in steps to higher beam energies (deeper penetration depths), differential depth resolved cathodoluminescence spectroscopy (DDRCLS) can be used to remove spectrum contributions from lower beam energies, enabling higher depth resolution. Another significant advantage of DRCLS is the keV beam energy range, which is low enough to be nondestructive so that the battery interface can be studied without introduction of new defects, altering the existing structure, or damaging the battery, but is still high enough energy to probe near 200 nm into this layered battery structure.

**Figure 1:**
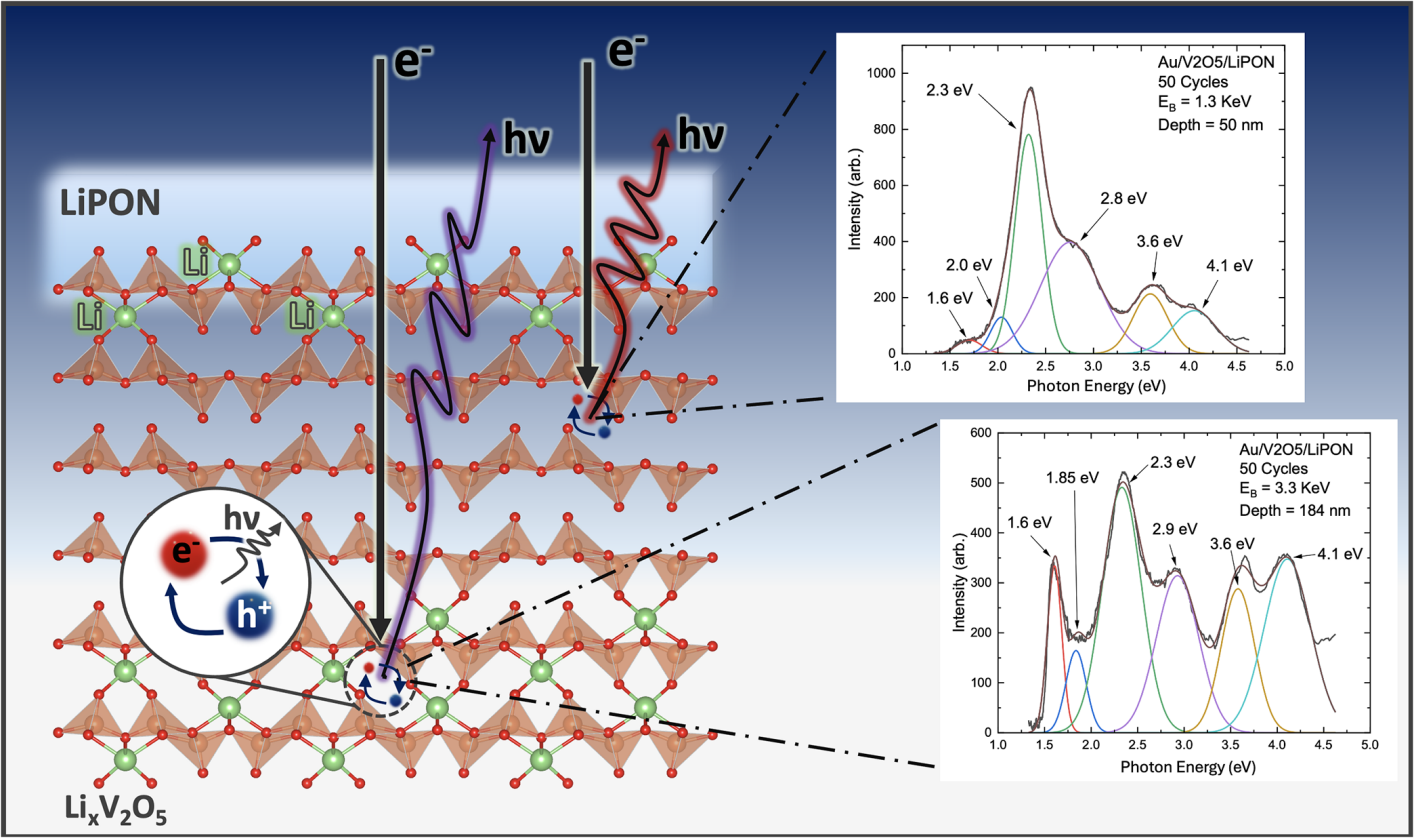
Schematic of electron beam incident on sample consisting of LiPON on top of Li_*x*_V_2_O_5_. Corresponding spectra (right) show one scan taken close to interface (top) and one scan taken deep into the bulk of the Li_*x*_V_2_O_5_ (bottom) showing the growth of an additional feature extending into the Li_*x*_V_2_O_5_ now identified as a midgap defect with a ∼1.6 eV peak signature.

The exact penetration depth of the electron beam can be estimated by using a Monte Carlo simulation.^17^ In our DRCLS simulations, initial beam currents penetrate materials with specific densities, atomic weights, and thicknesses to produce typically 2000000 incident electrons. The simulation produces a depth distribution reached by these forward and backscattered electrons, which provides the predicted penetration depth of that beam energy for that specific sample. These Monte Carlo simulations then yield penetration depths which increase with increasing E_B_, enabling nanometer-scale tuning of the depth below the outer surface within samples. This means that the interface can be probed directly and nondestructively, a capability previously unavailable when studying buried battery interfaces.

DRCLS is applicable to solid-state battery systems in which electrically active point defects produce luminescence through electron–hole pair recombination within the band gap.^18^ This includes many oxide-based and some sulfide-based electrolytes and electrodes (e.g., transition-metal oxides/sulfides, argyrodites, perovskites), where active defects, such as oxygen vacancies, give rise to strong characteristic luminescence signatures. However, an important limitation to DRCLS regarding solid state battery analysis is that it is only useful when studying materials with sufficient luminescence efficiency and optically active defect states. Materials with low band gaps, strong nonradiative recombination, or high electron-beam sensitivity can yield weak or ambiguous signals, making DRCLS analysis less effective. This is especially relevant to some sulfide-based electrolytes, as sulfide electrolytes tend to have relatively small bandgaps compared to oxide-based electrolytes.^19^

DRCLS offers several advantages over other analysis methods, such as XPS and electron paramagnetic resonance (EPR), in studying buried interfaces in solid-state materials. The most important advantage is that DRCLS is inherently nondestructive, unlike XPS depth profiling. Depth resolution in DRCLS is achieved by tuning the electron beam energy rather than by ion sputtering, thereby avoiding preferential sputtering, beam-induced reduction, and artificial defect formation that can significantly affect sample chemistry during XPS etching.^20^ While, similarly to DRCLS, EPR is a nondestructive technique, EPR can only detect paramagnetic centers and provides bulk-averaged information.^21^ In contrast, DRCLS is sensitive to both paramagnetic and diamagnetic defects that produce radiative recombination and offers nanoscale depth resolution across layered heterostructures. The ability to probe layered heterostructures nondestructively is significant in understanding how defects propagate in and away from electrolyte/electrode interfaces.

## Experimental Section

To fabricate the LiPON/Li_*x*_V_2_O_5_ samples, 3 in. p-type Si wafers were coated with 500 nm SiO_2_ in a Tystar chemical vapor deposition (CVD) furnace. A 10 nm Ti adhesion layer and 100 nm Au current collector were sputtered on the SiO_2_ substrate via a shadow mask to fabricate an array of devices within the 3 in. diameter wafer. RF sputtering was used to deposit 150 nm of V_2_O_5_ to serve as the cathode. A V_2_O_5_ target was sputtered at 153 W, at a pressure of 2.5 mTorr under a 11:1 sccm of Ar:O_2_ at room temperature. The electrode step is followed by 40 nm of lithium phosphorus oxynitride (LiPON) deposited by a reactive sputtering process using a lithium phosphate target (Li_3_PO_4_) with a power of 65 W under a 3 mTorr N_2_ atmosphere. After the LiPON deposition, the entire stack was post annealed at 300 °C for 3 h in 20 sccm N_2_.

Electrochemical cycling was performed using cyclic voltammetry (CV) in a beaker cell with LiClO_4_ in propylene carbonate (PC) at 1 M concentration. The LiPON film prevents Li-ions moving from the electrolyte directly to the V_2_O_5_, greatly minimizing flux and undesired reactions.^22^ These experiments were performed inside an argon glovebox with the beaker cell connected to a BioLogic potentiostat. Using a Li–metal counter/reference electrode and LiPON/Li_*x*_V_2_O_5_ as the working electrode, the devices were cycled between 4.0 and 2.2 V, starting with the lithium insertion step to 2.2 V and ending at 4.0 V on the last cycle at a rate of 0.5 mV/s. Electrochemical impedance spectroscopy (EIS) was taken throughout the cycle life of the samples. During each EIS measurement, the sample was held at 4.0 V for 90 s before continuing the CV cycling. Samples were thoroughly rinsed in PC to remove excess electrolyte and SEI deposits, and vacuum-dried at room temperature before being sealed under Ar atmosphere and transferred to Ohio State for DRCLS.

Samples were loaded into an ultrahigh vacuum chamber in preparation for DRCLS measurement. During measurement, a glancing incident PHI 05-045 electron gun and beam controlled by a PHI 11-010 electron gun controller impinged on the LiPON/LVO sample, causing a cascade of secondary electrons. When these electrons recombined, the light emitted was collected by a quartz lens and passed through a sapphire window into an Oriel 260i monochromator. The intensity and wavelength distribution of the CL signal was then recorded by an Andor DV420-OE CCD to produce the raw spectra for analysis. Raw spectra were finally deconvolved to identify characteristic and defect peak energies and intensities.

Plane wave density functional theory (DFT) calculations were carried out using the Vienna ab initio simulation package (VASP),^23,24^ with the generalized gradient approximation (GGA) exchange–correlation function of Perdew, Burke, and Ernzerhof (PBE).^25^ A *U*-value of 3.25 was used for vanadium to correct for the *d*-electron self-interactions, in order to give reasonable oxygen vacancy formation energies and electronic structures. The electronic structure of V_2_O_5_ with an oxygen vacancy correlates well with the work of Scanlon et al. where a *U*-value of 4 was used.^26^ An energy convergence criterion of 10^–6^ eV was used for each electronic step, while 0.01 eV/Å was used for the force convergence criterion. Additionally, a cutoff energy of 500 eV and a Gaussian smearing of 0.1 eV were utilized for all calculations. Both oxygen vacancy and nudged elastic band (NEB)^27^ calculations were performed on a 1 × 3 × 3 supercell with 126 atoms, a 3 × 1 × 1 supercell with 96 atoms, and a 3 × 1 × 1 supercell with 108 atoms for the α-V_2_O_5_, δ-LiV_2_O_5_, and γ-Li_2_V_2_O_5_ phases, respectively. Structure relaxations were conducted with a 2 × 2 × 2 Monkhorst–Pack *k*-point mesh, whereas a reduced 1 × 1 × 1 k-point mesh was used for NEB calculations.

Point defect calculations, including oxygen/lithium vacancies and interstitials, were screened on all the possible sites following the charged point defects DFT calculation framework,^28,29^ where the defect formation energy *E* ^f^[*X* ^*q*^] of defect *X* with charge *q* is defined as

1 $$E^{f}\left[X^{q}\right]=E\left[X^{q}\right]-E\left[\mathrm{bulk}\right]-\underset{i}{\sum }n_{i}\mu_{i}+{qE}_{F}$$

The *E*[*X* ^*q*^] and *E*[bulk] are the DFT calculated energies of the defect supercell and pristine bulk, respectively. μ_*i*_ is the chemical potential of species *i*, while *n*_*i*_ represents the number of units of species *i* added/removed to create a given defect. *E*_F_ is the Fermi level. The procedure, along with the correction energy, is automated by the doped Python package.^30^ The defect site fraction *X*[*X* ^*q*^] and concentration *c*[*X* ^*q*^] can then be computed—where *N* is the total number of sites for a given species—from the defect formation energy as

2 $$X\left[X^{q}\right]=\frac{c\left[X^{q}\right]}{N}=\exp\left(-\frac{E^{f}\left[X^{q}\right]}{k_{B}T}\right)$$

By imposing charge neutrality of all point defects, including electrons and holes, the Fermi-level *E*_F_, is obtained.^31^ The defect formation energy and concentration at charge neutrality are then determined at this Fermi level. The details of these defect calculations are presented in,^32^ here only the lowest energy oxygen vacancy results are presented to compare with experiments.

## Results

Previous studies^33–35^ have shown that Cr in materials such as Al_2_O_3_ or Ga_2_O_3_, whether introduced intentionally or unintentionally as a defect, can act as an effective optical marker in luminescence spectroscopy studies. This is because, when introduced into these materials, a Cr^3+^ optical center is formed. Specifically, in Ga_2_O_3_ the Cr^3+^ substitutes for Ga at the octahedral Ga­(II) site. Spin–orbit coupling and the crystal field cause the splitting of the 3d^3^ orbital of Cr^3+^ into multiple sublevels. Specifically, two absorption band transitions (^4^A_2_ → ^4^T_2_ and ^4^A_2_ → ^4^T_1_) and a doublet emission transition (^2^E → ^4^A_2_) are typically observed. The latter produces two sharp luminescence lines, commonly referred to as the R_1_ and *R*_2_ peaks. These R_1_ and R_2_ peaks are typically detected at approximately 696.6 nm (∼1.78 eV) and 690 nm (∼1.80 eV). These R_1_/R_2_ split peaks form the basis for optical characterization of Cr^3+^-doped materials and are frequently used as spectral fingerprints in luminescence spectroscopy.

For this study, a very small percentage (∼2% of a single monolayer) of Cr was deposited at the interface between LiPON and Li_*x*_V_2_O_5_ to be used as an interfacial marker. DRCLS with beam energy increasing in 0.1 keV steps was then done until two sets of the Cr R_1_/R_2_ doublet peaks were observed. The two sets of doublet peaks indicated that the Cr emission was coming from recombination from both the LiPON and Li_*x*_V_2_O_5_ layers, indicating that the penetration depth of the beam was at exactly the interface. The identification of the interface led to fine-tuning of Monte Carlo simulations by adjusting the specimen tilt angle until the simulation penetrated to the interface at exactly the beam energy where the Cr R_1_/R_2_ peaks were detected. The Cr was removed from the sample design recipe after the Monte Carlo calibration was complete. As a result, the DRCLS data presented in this study was taken on samples that did not contain Cr.

V_2_O_5_ undergoes phase changes as it is lithiated.^12^ Phases of Li_*x*_V_2_O_5_ include α (0 < *x* < 0.05), ε (0.05 < *x* < 0.5), δ (0.5 < *x* < 1), and γ (1 < *x* < 2). The insertion of one lithium has a theoretical capacity of 147 mAh/g of V_2_O_5_, while insertion of two lithium has a theoretical capacity of 294 mAh/g of V_2_O_5_, however this is difficult to achieve in practice due to the large crystallographic distortions required to transform between the δ and γ phases. Poor Li extraction kinetics are commonly observed in γ-LVO.

CV was chosen for this study in order to limit local electrochemical overpotentials inhomogeneities, which could cause a distribution of LVO phases in the sample. However, since we control the voltage sweep rather than the amount of charge passed (as in galvanostatic cycling), reaction kinetics and diffusion rates can limit the quantity of charge inserted or removed on each cycle. Hence, our highest reported capacities are a fraction of the theoretical capacities listed above.

Cycling data is shown over 50 cycles in Figure 2a and demonstrates good reversibility of the Li reactions between 3.0 and 3.6 V (phases α > ε > δ), while between 2.0 and 2.4 V (δ > γ phase transition) insertion occurs quicker than Li deinsertion, as evidenced by the lack of any strong oxidation peak in this range. (Two additional, stable peaks between 2.5 and 2.75 V are ascribed to a VO_2_-like suboxide phase, which seems to have been produced during fabrication.) For this study, all reported potential windows are referenced to V_2_O_5_ vs Li/Li^+^. This choice is crucial as referencing Li to Au risks plating Li on the Au surface. By referencing potential exclusively to V_2_O_5_ vs Li/Li^+^ and staying above Li-plating potential of 1.0 V, we prevent any undesired side reactions.^36^

**Figure 2:**
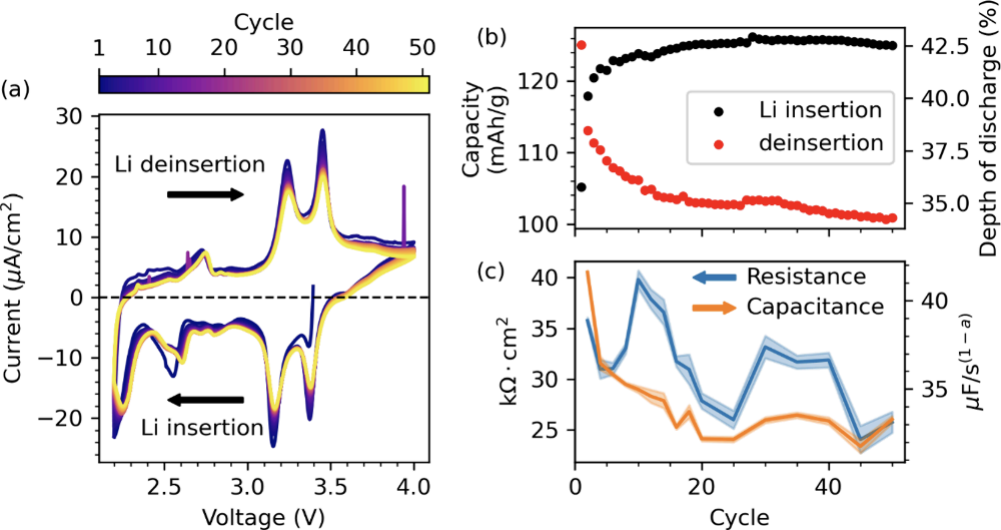
Electrochemical cycling and capacity of LiPON/Li_*x*_V_2_O_5_ devices. (a) Cyclic voltammetry (CV) at 50 cycles in a liquid beaker cell. (b) Electrode specific capacity (normalized by mass of V_2_O_5_) and depth of discharge as calculated by integrating the area under the CV curves. (c) Charge transfer resistance and capacitance parameters for the LiPON/LVO interface calculated from electrochemical impedance spectroscopy data. Shaded bands represent one standard deviation in the uncertainty of the fitted values.

Cycling through LVO electrochemical phases typically affects the capacity of the electrode as degradation occurs. The capacity shown in Figure 2b illustrates a nonmonotonic drop on each cycle. Li deinsertion experiences an 11% decrease from 112 mAh/g on the second cycle to just over 100 mAh/g. Li insertion starts around 120 mAh/g with about 6% increase early on, then later plateaus after about 20 cycles. As such we are inserting more Lithium into the LiPON/Li_*x*_V_2_O_5_ system than we are extracting. The Coulombic efficiency (ratio of discharge to charge capacity) is 104% on the second cycle and rises to more than 120% by the 15th cycle.

Nyquist plots of the EIS are shown for the 50 cycle sample in the SI, along with the equivalent circuit model used to fit the spectra and derive charge transfer resistance and capacitance of the LiPON/LVO interface,^37,38^ shown in Figure 2c. The charge transfer resistance is related to the equilibrium reaction rate at that cell potential, while the interface capacitance is ascribed to dipoles at the interface due to unbalanced charge (Li^+^, e^–^, or V_O_^+^). Since 4.0 V is far from any redox reactions, we expect the charge transfer resistance to be large and it generally trends down over 50 cycles, from around 35 kOhm.cm^2^ to less than 30, but with some variation. The capacitance, however, shows a smooth trend, dropping rapidly in the first 20 cycles before stabilizing for the last 30. This trend in capacitance is mirrored by the trend in cell capacity.

DRCLS was measured at various depths throughout the LiPON/LVO samples, including shallow scans in the LiPON layer, at the interface, and throughout the LVO bulk. The DRCLS spectra were then deconvolved to reveal characteristic peaks of Li_*x*_V_2_O_5_, as well as an additional feature shown in Figure 1 identified as a midgap defect with a ∼1.6 eV peak signature, which DFT calculations, presented below, identify as the contributions from an oxygen vacancy.

Initially, DRCLS on uncycled pristine samples showed a small V_O_ peak localized within the first 20 nm below the interface between the LiPON and V_2_O_5_ layers. This localized behavior indicates that the oxygen vacancy defect is tied to lithium insertion since our previous studies showed that Li ions diffuse up to 30 nm into Li_*x*_V_2_O_5_ from a LiPON overlayer.^15^ When Li-ions insert into the Li_*x*_V_2_O_5_ layered lattice, they preferentially occupy interlayer sites—coordinating with 8 O atoms in α-V_2_O_5_ via ionic bonds—which reduces the nearby V atoms. The weakened V–O framework can result in oxygen displacement from the pristine lattice sites, producing V_O_ that stabilizes the reduced vanadium states (V^5+^→V^4+^→V^3+^), however, the trend is not monotonic. In α-V_2_O_5_, the formation energy of an oxygen vacancy is 0.31 eV, which increases to 0.70 eV for δ-LiV_2_O_5_ then decreases to 0.16 eV for the fully lithiated γ-Li_2_V_2_O_5_. These point defect calculations show that Li intercalation can lower the formation energy of oxygen vacancies, making them more thermodynamically favorable at very high lithium contents. However, our previous DRCLS studies of lithiated Li_*x*_V_2_O_5_ samples without a LiPON overlayer showed no ∼ 1.6 eV defect peak.^12^ This indicates that the formation of oxygen vacancies in the V_2_O_5_ layer is tied to lithium ions moving in and out of the LiPON overlayer, not just lithiation of the V_2_O_5_ layer.^12^

### Modeling Confirmation of the 1.6 eV Oxygen Vacancy Peak

Previous literature^7–10^ had assumed charge-neutral oxygen vacancies (*v*_O_^••^), meaning each oxygen vacancy will reduce two V ions in the lattice. Without this preassumption, our defect calculations—which screened oxygen vacancies with 0, + 1, and +2 charges—yield the oxygen vacancy (*v*_O_^•^) reducing only one nearby V to be the most stable defect. When oxygen vacancies are referenced in this paper the notation V_O_ is used to denote the positively charged *v*_O_^•^ defect. Figure 3 shows the relaxed defect structure for each LVO phase, and the partial density of states (PDOS) of the *d*-orbitals on the reduced V.

**Figure 3:**
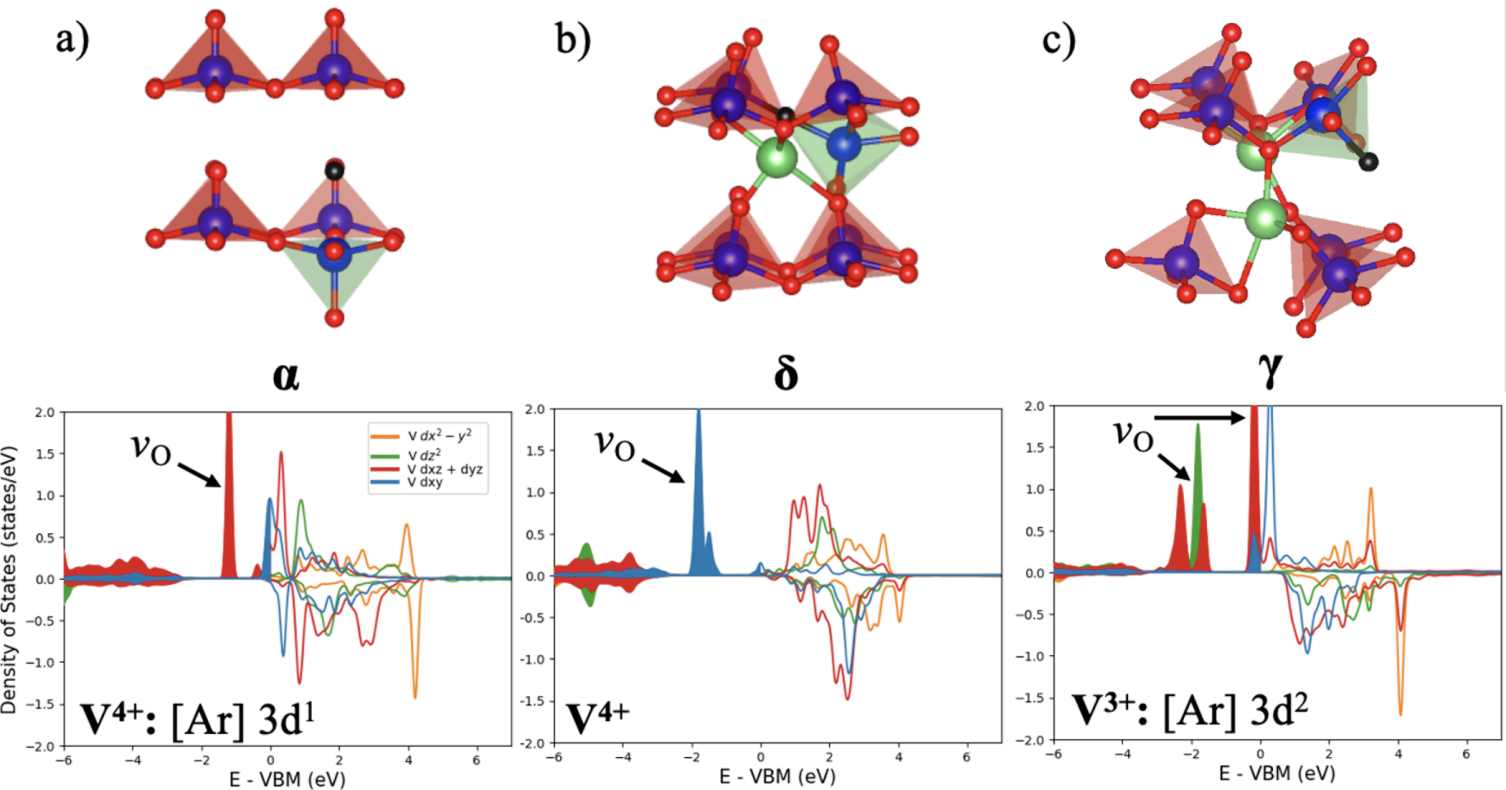
Relaxed local structures of the oxygen vacancy and *d*-orbital PDOS of V in LVO, with an oxygen vacancy. (a) α-V_2_O_5_, (b) δ-LiV_2_O_5_, and (c) γ-Li_2_V_2_O_5_. Colors: blue atom, V in the lattice; green polyhedron, V connected to the oxygen vacancy; black atom, oxygen vacancy; red atom, oxygen in the lattice; green atom, lithium.

For α-V_2_O_5_, the oxygen coordinated to only one V, referred to as the O1 oxygen in the literature, is the most stable defect, consistent with previous DFT calculations.^9^ The nearby reduced V^4+^ (green polyhedron in Figure 3a) drops to the middle of the layer and tries to bond with an oxygen atom in the next layer. Due to the easy reduction of V^5+^ in pristine V_2_O_5_, the oxygen vacancy formation is 0.31 eV. The reduced V^4+^ indeed shows an additional midgap state nearly 1.6 eV above the valence band maximum (VBM). In δ-LiV_2_O_5_, half of the vanadium is V^4+^ and half is V^5+^. The presence of an oxygen vacancy creates an additional V^4+^ that produces a distinct, narrower state near the VBM as compared to the native V^4+^ that arise due to the increased Li^+^ content. The reduced V^4+^ next to the oxygen vacancy also drops into the Li layer. Due to the repulsion of the Li and V in the same layer, the oxygen vacancy formation energy is 0.70 eV, much larger than the previous phase. Lastly, in γ-Li_2_V_2_O_5_, all V^5+^ are reduced to V^4+^. Creating an oxygen vacancy will reduce one V^4+^ to V^3+^, which did not show large displacements. Comparing the PDOS of V^3+^ with that of V^4+^, formed due to Li insertion, the d_z2_ orbital is filled, at −2 eV below the VBM. This new state arises since the V hosting the oxygen vacancy cannot move in the lattice to form an additional bond with neighboring O, due to the high Li^+^ content in γ-Li_2_V_2_O_5_. This leaves the V host coordinated to only four O, resulting in a square planar bonding environment, which causes the d_*z* ^2^_ orbital to drop down in energy. The oxygen vacancy formation energy in γ-Li_2_V_2_O_5_ is 0.16 eV, likely due to the smaller V displacement. Overall, these results confirm that the new 1.6 eV peak found in the DRCLS measurements is due to the reduced and displaced V^4+^ next to the oxygen vacancies in α-V_2_O_5_ and δ-LiV_2_O_5_; and it is likely that the d_z2_ peak and the peak near the VBM in γ-Li_2_V_2_O_5_ also contribute to this emission. Based on the oxygen vacancy formation energy, the oxygen vacancy concentrations are 2.4 × 10^17^ cm^–3^ for α-V_2_O_5_, 1.0 × 10^16^ cm^–3^ for δ-LiV_2_O_5_, and 1.3 × 10^18^ cm^–3^ for γ-Li_2_V_2_O_5_. As a fraction of all possible oxygen positions within each lattice, these values relate to an oxygen vacancy concentration of less than 1% for each phase. Because of the nonmonotonic change of oxygen vacancy concentration with Li-concentrations, the phase change may lead to trapped oxygen vacancies at phase boundaries corresponding to the spatial movement of oxygen vacancies peak across the LiPON/LVO interface.

Next, DRCLS was measured on samples after 5, 10, 20, and 50 electrochemical cycles. Figure 4 shows 50 cycle Li_*x*_V_2_O_5_ spectra from the sample’s outer LiPON layer to depths extending into the Li_*x*_V_2_O_5_ layer bulk. We find that the ∼1.6 eV peak is present at all depths, growing in intensity with increasing depth into the bulk. This contrasts with previous DRCLS, where no 1.6 eV peak is present even in lithiated Li_*x*_V_2_O_5_ samples without a LiPON overlayer.^12^ The only other reported midgap state in V_2_O_5_ was at ∼0.8 eV in a PL study of the free V_2_O_5_ surface and attributed to oxygen vacancies.^5^ However, the 1.6 eV emission feature shown in Figure 4 matches the DFT calculations predicting a midgap oxygen vacancy feature at ∼1.6 eV. To experimentally confirm the oxygen vacancy nature of the 1.6 eV emission, we oxygen annealed then cycled samples for comparison with unannealed samples. The reduction of 1.6 eV emission with oxygen annealing, shown in Figure S3, confirmed its relation to oxygen vacancies.

**Figure 4:**
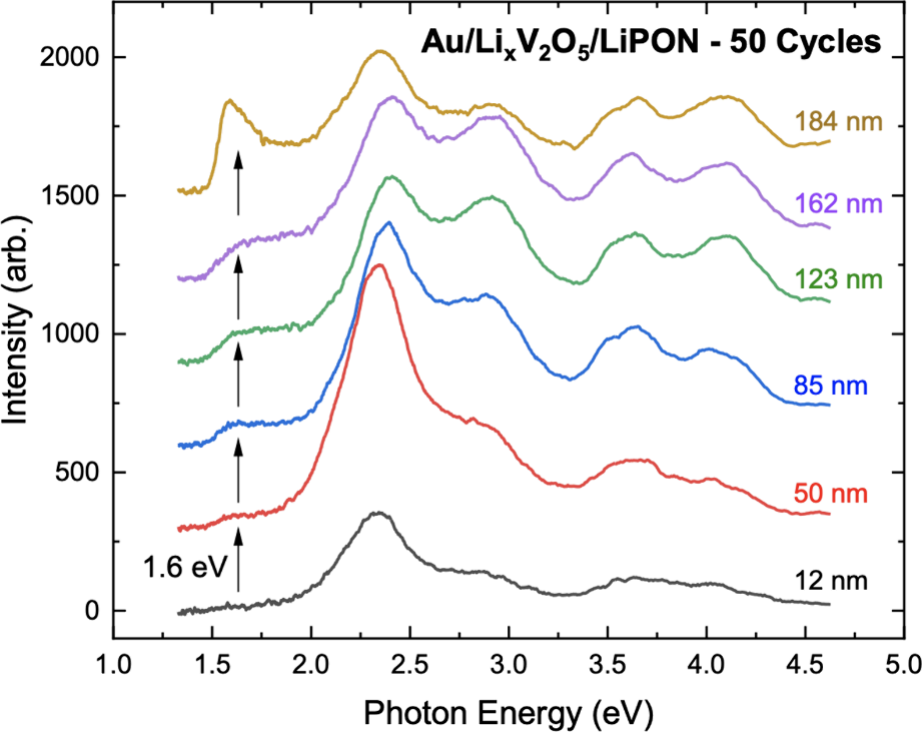
DRCLS spectra from Au/Li_*x*_V_2_O_5_/LiPON sample after 50 continuous cycles. Spectra show a clear defect peak at 1.6 eV, which is now identified as an oxygen vacancy. This V_O_ emission clearly increases in intensity as electron beam probes deeper into the sample.

Figure 4 also shows increasing intensities with increasing depth of three other peaks (2.9, 3.6, and 4.0 eV) in addition to the 1.6 eV peak. The 3.6 and 4.0 eV are characteristic V_2_O_5_ peaks that are present in α-V_2_O_5_, δ-V_2_O_5_, and γ-V_2_O_5_. Generally, the 3.6 eV peak is strongest in in α-V_2_O_5_, while the 4.0 eV peak is strongest in δ-V_2_O_5_,^12^ Since both peaks are increasing in intensity with increasing depth, this likely indicates a mixing of V_2_O_5_ phases as we probe deeper into the sample as neither peak strongly dominates compared to the other, nor does one peak increase more than the other. The increase in the ∼2.9 eV peak may also indicate a mixing of phases as it is quite broad and could be encapsulating both the 2.45 eV feature from δ-V_2_O_5_ and the ∼3.1 eV peak from both the α-V_2_O_5_ and the δ-V_2_O_5_ phases. Neither of these features produce significantly sharp peaks,^12^ so it is possible that in the case of phase-mixing, a broad energy-shifted peak could appear as a combination of the two, such as the broad peak centered at ∼2.9 eV.

After detecting the ∼1.6 eV defect and confirming with theory and experiment that it matches the energy predicted for the midgap oxygen vacancy peak, DRCLS measurements focused on understanding how oxygen vacancies peak grow as a function of battery cycling. In general, it is known that batteries degrade as they are continually cycled, but the mechanisms causing this degradation are not well understood. Observation of how the oxygen vacancy defect peak changes over the lifespan of a battery device structure could provide an explanation for a key degradation mechanism.

As mentioned above, the samples were cycled to various stages in a battery’s lifespan, ranging from pristine to 50 cycles. DRCLS was performed on each sample (5, 10, 20, and 50 cycles) and the intensity of the 1.6 eV oxygen vacancy peak, normalized to the total intensity of each spectrum, was tracked as a function of depth. Figure 5 shows a depth profile of the 1.6 eV V_O_ peak, where the normalized intensity of the 1.6 eV defect peak was tracked as a function of beam penetration depth. The total oxygen vacancy feature intensity throughout all depths grows as the cycle number increases, although the maximum intensity at a singular depth occurs at ∼85 nm for the 10 cycle sample. Furthermore, the position of the maximum intensity of the oxygen vacancy peak moved further away from the LiPON/Li_*x*_V_2_O_5_ interface with increasing cycle number. This indicates that cycling has a considerable effect on the development of oxygen vacancy defects.

**Figure 5:**
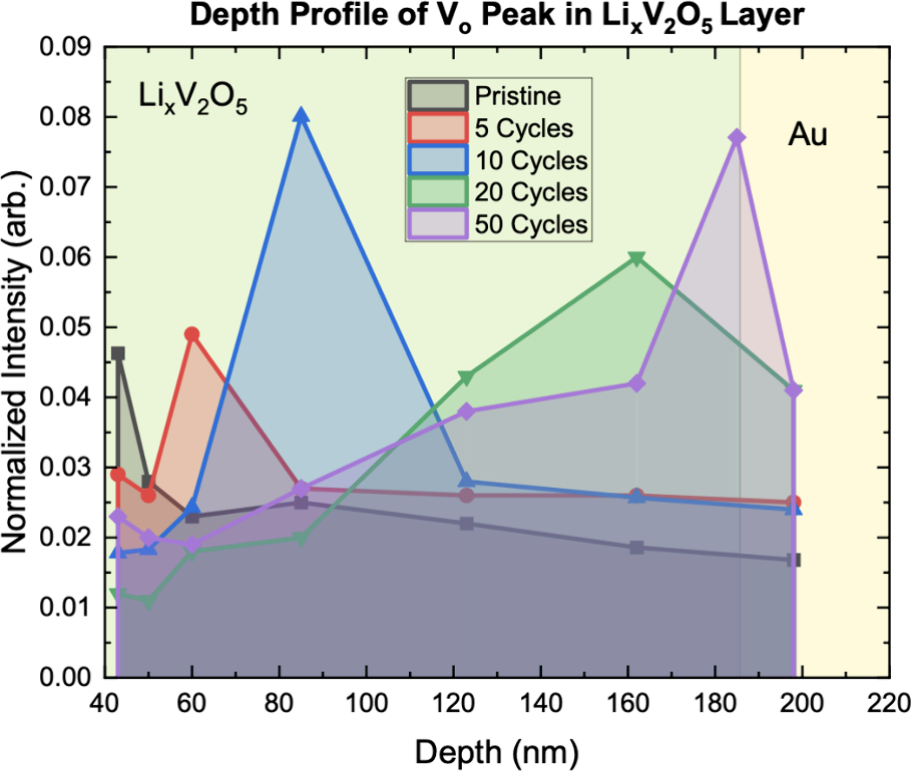
Depth profile of 1.6 eV oxygen vacancy peak in Li_*x*_V_2_O_5_ for each cycled sample as a function of depth. The figure shows increasing oxygen vacancy intensity as well as V_O_ defect movement further into the bulk with increasing cycle number.

The depth profile can also inform how many total oxygen vacancies there are in the sample. From the depth profiles in Figure 5 showing the normalized intensity of oxygen vacancies as a function of depth at each cycle number, the area under each sample’s respective depth profile curve is integrated, giving the relative amount of oxygen vacancies throughout the Li_*x*_V_2_O_5_ layer. Figure 6 shows that the amount of oxygen vacancies continually increases as cycle number increases. A strong increase in V_O_ amount appears between uncycled and 10-cycled-LVO before the changes become significantly less.

**Figure 6:**
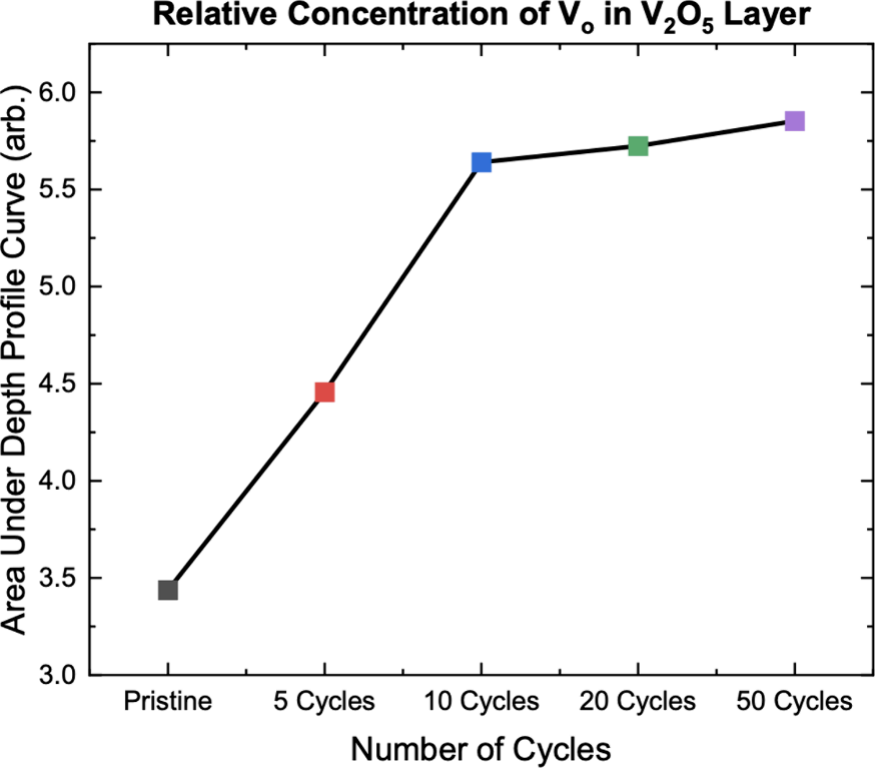
Relative total concentration of oxygen vacancies with increasing cycle number, created by taking the area under the curve for each cycle number from Figure 5, with the data markers matching the respective curve colors. Oxygen vacancy concentration clearly increases as cycle number increases.

This behavior indicates that oxygen vacancies increase with cycling and that the very early cycles play a major role in this defect evolution. Furthermore, the slight plateau in oxygen vacancies past 10 cycles could indicate that an oxygen vacancy saturation occurs, which might be consistent with how oxygen vacancies are created through lithiation induced phase changes as described above, possibly limiting the number of oxygen vacancies that can be formed in the Li_*x*_V_2_O_5_.

### Device Degradation via Oxygen Vacancy Formation and Accumulation

LVO can reversibly be cycled up to 2 Li^+^ per V_2_O_5_ formula unit. However, previous studies^39^ have shown that repeated ion insertion can lead to substantial structure rearrangements and destabilization, ultimately leading to device failure. Additionally, computational methods^8,10^ have shown that the inclusion of oxygen vacancies into the LVO lattice increases the diffusion barrier for Li^+^ in the pristine α-V_2_O_5_ and the fully delithiated γ-V_2_O_5_, therefore degrading the rate capacity.

However, other groups^40,41^ have reported that the inclusion of oxygen vacancies is likely to increase the performance of the cathode since they will donate electrons to the system and will enhance the electronic conductivity of the cathode. Therefore, the role of oxygen vacancies in the performance of LVO cathodes remains an open question. Through a combination of DFT calculated point defect formation energies and nudged elastic band (NEB) diffusion barrier calculations, we deconvolved the impact of oxygen vacancies to show that they are a key factor contributing to device degradation through destabilization of the cathode structure and trapping of Li^+^.

#### Diffusion Barrier Calculations

To maintain good cyclability, the diffusion barrier for Li^+^ should remain small inside the cathode during each stage of lithiation and delithiation. To track the effect of oxygen vacancies, a series of six NEB calculations were carried out on three orthorhombic phases of LVO, namely: α-V_2_O_5_, δ-LiV_2_O_5_, and γ-Li_2_V_2_O_5_. For each phase, the diffusion barrier for Li^+^ was calculated (i) in the pristine phase, and (ii) in a supercell containing a single oxygen vacancy. The energy along the reaction coordinate for each Li^+^ diffusion path are shown in Figure 7.

**Figure 7:**
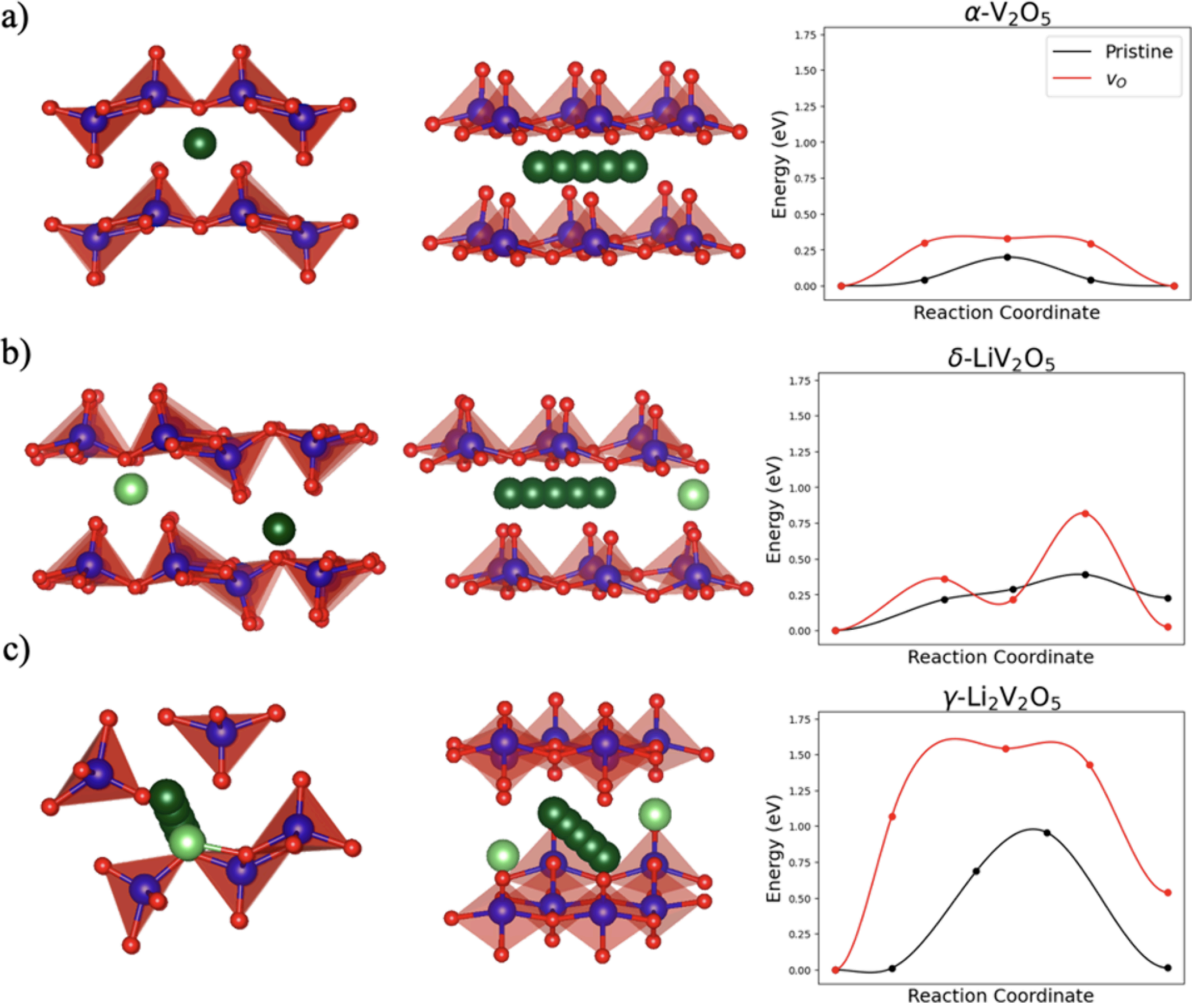
Structures and NEB diffusion barriers for (a) α-V_2_O_5_, (b) δ-LiV_2_O_5_, and (c) γ-Li_2_V_2_O_5_. The dark green atoms represent the Li^+^ diffusion path, while light green atoms denote additional Li at equilibrium positions, blue atoms are V, and O are shown in red. Two views of the same diffusion path are presented, the first showing the position of the path between the Li_*x*_V_2_O_5_ layers, while the second shows the intermediate positions Li takes between two equilibrium sites.

For each phase, Li^+^ diffusion barriers were calculated only along the channel created by adjacent Li_*x*_V_2_O_5_ layers since previous work^10^ has identified this as the most favorable direction for diffusion. These calculations reveal two important trends: first, that the Li^+^ diffusion barrier increases as the Li content increases in LVO, and second, that the inclusion of an oxygen vacancy in the LVO lattice increases the diffusion barrier for all three phases.

For the α-V_2_O_5_ phase, the pristine diffusion barrier was calculated to be 0.18 eV, in good agreement with previous results.^8,10,42^ Upon lithiating to δ-LiV_2_O_5_, the diffusion barrier increases to 0.33 eV. This large increase is most likely due the repulsion between adjacent Li^+^ intercalated in the LVO lattice. Fully lithiating to the γ-Li_2_V_2_O_5_ phase results in a prohibitively high diffusion barrier of nearly 1.0 eV.

When γ-Li_2_V_2_O_5_ is reached, two effects, both caused by lithiating past 1 Li^+^ per V_2_O_5_ per unit cell, couple to create such a high diffusion barrier. First, once δ-LiV_2_O_5_ is reached, Li^+^ fill all the available 2b Wyckoff positions in the lattice. Therefore, to accommodate more than 1 Li^+^ per formula unit, an additional Wyckoff position, namely, the 4c position, has to be filled. This decreases the Li–Li distance by nearly 1 Å, increasing the repulsion between the ions which results in a more arduous diffusion pathway. Second, such a high degree of lithiation severely puckers the V_2_O_5_ layers, leading to a constriction of the diffusion channel and less room for Li^+^ to diffuse. The high diffusion barrier present in γ-Li_2_V_2_O_5_ is likely to lead to sluggish kinetics and contribute to the poor rate capacity of the LVO cathode due to Li^+^ becoming essentially trapped within the cathode.

Following the red curves in Figure 7, it is clear that the inclusion of oxygen vacancies further degrades the sluggish kinetics seen at higher degrees of lithiation. An oxygen vacancy at the O1 position was incorporated into each LVO phase as close to the diffusing Li^+^ as possible, this position will be referred to as the nearest-neighbor oxygen vacancy. For α-V_2_O_5_ the inclusion of a nearest-neighbor oxygen vacancy causes the diffusion barrier to nearly double by inducing a local shrinking of the diffusion channel by nearly an angstrom. This occurs since the V^4+^ ion hosting the oxygen vacancy shifts into the diffusion channel, in order to form a bond with an oxygen from the adjacent V_2_O_5_ layer.

In δ-LiV_2_O_5_, the diffusion barrier again doubles for the supercell with a nearest-neighbor oxygen vacancy as compared to the pristine case. The rationale for this large increase is similar to the previous phase: the V^4+^ ion hosting the oxygen vacancy puckers out into the diffusion channel introducing an additional obstacle for Li^+^ diffusion.

The increase in the diffusion barrier is also pronounced in γ-Li_2_V_2_O_5_, where the barrier rises to 1.50 eV. Due to the large Li^+^ content in the γ-Li_2_V_2_O_5_ phase, the V^3+^ hosting the oxygen vacancy cannot move to form an additional oxygen bond; this leaves the diffusion channel largely unchanged and results in a smaller relative increase in the diffusion barrier as compared to oxygen vacancy incorporation in the other two phases.

For completeness, oxygen vacancies were also included in the lattice at the furthest possible position from the diffusing Li^+^ to see if the effect on the diffusion barrier persists at longer distances. The faraway oxygen vacancies did increase the diffusion barrier, but by a smaller amount than the nearest-neighbor oxygen vacancies.

Additionally, we tested the possibility of coupled diffusion between Li^+^ and the oxygen vacancies to determine if concerted motion of these two species would lower the diffusion barriers for Li^+^. For each phase, two concerted diffusion paths were tested, one with a stationary Li^+^ that an oxygen vacancy diffuses past, and a second path with the oxygen vacancy diffusing first followed by a Li^+^ diffusing behind it. All the concerted motion diffusion paths resulted in higher barriers as compared to the single species diffusion paths.

It is plausible that oxygen vacancies can lead to more electrons in the lattice; however, their impact on electric conductivity may be small, as LVO is already a good n-type or p-type electronic conductor based on DFT calculations. Importantly, our results show that the concentration of oxygen vacancies will grow inside LVO, and furthermore, that the inclusion of oxygen vacancies in the LVO structure increases the Li^+^ diffusion barrier, which traps Li^+^ and leads to irrepressible capacity loss. These effects will degrade capacity and performance.

Finally, since the lithiation of LVO proceeds via phase segregation, there may be additional kinetic barriers for Li^+^ to transfer from less lithiated to more lithiated regions or vice versa. However, the NEB calculations describe the general trends that higher Li-contents and the presence of O-vacancies will both slow Li^+^ diffusion. While additional diffusion barrier calculations would help to calculate the actual diffusivity of Li^+^ in LVO, as Li^+^ diffusion will be depended on the local environment (Li and O-vacancy content), these trends are likely to remain the same and lead to the same conclusion, namely that more Li and O-vacancies lead to reduced diffusion and will degrade battery performance.

## Discussion

The DRCLS measurements provide experimental evidence for the creation and movement of oxygen vacancies in Li_*x*_V_2_O_5_ thin films interfaced with LiPON during electrochemical cycling. Initial measurements revealed a weak peak at 1.6 eV confined to the first 20 nm past the interface in the uncycled pristine samples. This observation is consistent with previous studies reporting lithium penetration up to ∼30 nm into Li_*x*_V_2_O_5_ films from a LiPON layer,^15^ where Li^+^ insertion destabilizes the V – O framework and reduces vanadium centers to V^5+^/V^4+^ states. Prior DRCLS studies on Li_*x*_V_2_O_5_ in the absence of a LiPON overlayer did not show the ∼1.6 eV feature,^12^ highlighting that the defect formation process is strongly influenced by Li-ion exchange across the LiPON interface, rather than simply bulk lithiation of V_2_O_5_.

The discovery of this peak is significant because it is the first spectroscopic measurement confirming DFT theory predictions of a 1.6 eV midgap oxygen vacancy feature. This contrasts with earlier photoluminescence studies, which only identified an oxygen vacancy state near ∼0.8 eV, only within the absorption depth of the light, and without any depth resolution to address buried interfaces. Furthermore, this DRCLS measurement was done nondestructively, meaning the battery did not have to be damaged to perform the measurement. This made a cycling-based study possible, as the interface and bulk of cycled batteries could now be probed without having to cleave or alter the sample.

Upon cycling, a distinct evolution of the ∼1.6 eV feature was observed. The relative maximum energy of the V_O_ peak increased with depth (as shown in Figure 5) and the overall intensity increased as cycle number increased (as shown in Figure 6). This indicates that oxygen vacancies not only increased in number with continued cycling but also migrated progressively into the Li_*x*_V_2_O_5_ bulk. Note that V_2_O_5_ was not fully recovered after each cycle, as indicated by the capacity loss. This behavior is also represented in the EIS-measured interface capacitance in Figure 2c. Since the EIS was collected in a nominally delithiated state, capacitance at the interface may be attributable to V_O_ on the LVO side. As the V_O_ migrates into the bulk, the interface capacitance drops, and the charge transfer resistance is seen to decrease as well. Combined with DFT simulations, which show that oxygen vacancies significantly increase the lithium diffusion barrier in LVO, it can be concluded that the aggregation of oxygen vacancies over a battery’s lifespan also contribute to significant loss in battery rate performance (lithium diffusion) and capacity. Therefore, oxygen vacancies likely play a key role in battery degradation over a battery’s lifespan by increasing the lithium diffusion barrier energy throughout the Li_*x*_V_2_O_5_ lattice.

In addition to the vacancy-related feature, analysis of the broader DRCLS spectra provides insight into how oxygen vacancy formation may influence Li_*x*_V_2_O_5_ phase stability. The shallow-depth spectra exhibited peaks at 2.0, 2.8, 3.6, and 4.1 eV, which seem to be more characteristic of δ-LiV_2_O_5_ phase features,^12^ while deeper scans revealed features at 1.85, 2.9, 3.6, and 4.1 eV consistent with α-V_2_O_5_ phase. These DRCLS features did not precisely match the previously reported energies for either α- or δ-LVO and thus cannot yet definitely confirm that these are the exact phases present. Nevertheless, the results clearly show changing peak energies in the sample as we probed deeper, indicating that some phase changes occurred, and these phases continue to grow further from the interface as additional residual Li accumulates after each cycle. More detailed comparison between Figure 4’s DRCLS peak features vs depth and the corresponding single α, δ, and γ phases of LVO depth profiles^12^ shows that, unlike Figure 4’s changes in peak energies, peak shapes, and the number of peaks as a function of depth, the single-phase depth profiles are uniform with depth (see Figure S4). Figure 4 shows features at ∼3.6 and ∼4.1 eV, which are common to all three phases, but they are accompanied by even stronger peaks at 2.6 and 2.9 eV. These 2.6 and 2.9 eV peaks suggest that some δ phase and possibly even some α phase may also be present. The evolution of all these peaks in Figure 4 suggests a mixture of phases that include γ at all depths, increasing strongly at 50–85 nm depths, then remaining high. These results suggest that oxygen vacancies will accompany the phase transitions during lithiation. As the oxygen vacancy formation is the easiest in the γ-LVO, the defect chemistry can play a critical role in determining the location of different phase of Li_*x*_V_2_O_5._ Such phase transformations are directly relevant to capacity fade, as the electrochemical properties of the α, δ, and γ phases of V_2_O_5_ differ substantially.

Figure 6 shows the total relative concentration of oxygen vacancies in the sample as a function of cycling, clearly showing that oxygen vacancy concentration increases as cycle number increases. However, there is a sharp contrast between the change in oxygen vacancies between 0 and 10 cycles and between 20 and 50 cycles. In the first 10 cycles, the oxygen vacancy concentration increases rapidly while the concentration increases much more slowly after 10 cycles, almost plateauing. This trend is very similar to the irreversible Li loss in Figure 2b. The plateau in the creation of V_O_ in Figure 6 is consistent with how oxygen vacancies form more easily in γ-Li_2_V_2_O_5_, which is also the irreversible phase.

While the data shows that oxygen vacancies clearly aggregate as the LiPON/Li_*x*_V_2_O_5_ sample is cycled, it is not clear where the oxygen atoms migrate to after being released through the vanadium reduction. One possibility is that these atoms migrate into the LiPON overlayer and out of the free surface. However, understanding this behavior in future studies could reveal much more about the degradation of the overall LiPON/Li_*x*_V_2_O_5_ system.

## Conclusion

The results in this paper show that oxygen vacancies emit a 1.6 eV signal, which DRCLS observed directly throughout the depth without the need to damage the LiPON/Li_*x*_V_2_O_5_ sample. This is significant, as it is the first time that the 1.6 eV midgap V_O_ peak predicted by theory has been confirmed. The nondestructive nature of DRCLS allowed a new method of investigating batteries that has not been previously employed and opens the door for further nondestructive studies of battery defects, such as in situ measurements during biasing/cycling.

Furthermore, the findings provide a mechanistic framework for understanding a previously underexplored degradation pathway in LiPON/Li_*x*_V_2_O_5_-based solid-state batteries. First, previous DRCLS results did not show the 1.6 eV oxygen vacancy peak when cycling V_2_O_5_, and DFT oxygen vacancy formation energies, computed in this work, correspond to low concentrations of the defects. Therefore, the growing intensity of oxygen vacancies measured by DRCLS comes from cycling in the presence of a LiPON overlayer; suggesting that LiPON acts as a source of oxygen vacancies. The incorporation of oxygen vacancies into LVO increases the Li^+^ diffusion barrier at all Li contents. Subsequent cycling of the LiPON/LVO interface leads to increased oxygen vacancy signal which further slows Li^+^ diffusion, effectively trapping Li in the LVO lattice as seen in the evolution of the DRCLS peaks which shows the presence of γ-Li_2_V_2_O_5_ at all depths. Finally, DFT shows that oxygen vacancies will form easiest in the γ-phase which can lead to further trapping of Li and additional capacity fade.

This multifaceted impact highlights the need to consider oxygen vacancy chemistry alongside lithiation dynamics when designing vanadium oxide cathodes and solid-state electrolyte interfaces. Future work should involve more cycling studies, such as investigation of defects as a complete battery structure is cycled to failure. We are now developing new lateral all solid-state batteries which enable DRCLS probing throughout the whole sample. These new battery structures will allow for in situ cycling studies while taking batteries completely to failure. These types of studies will also open avenues to other future work exploring whether defect engineering strategies, such as controlled doping, oxygen-rich processing, or interface modification, can suppress vacancy formation and thereby extend the lifetime of Li_*x*_V_2_O_5_-based devices.

## Supplementary Material



## Notes

Additional experimental details, including Monte Carlo simulations, electrochemical impedance spectroscopy plots, and V_2_O_5_ phase comparison (PDF)
