# Supplementary material for: Oxygen Vacancy Evolution at Li x V2O5/LiPON Solid State Electrochemical Interfaces Using Depth Resolved Cathodoluminescence Spectroscopy
Source: ACS Appl Mater Interfaces. 2026 Apr 17;18(16):23730–40. doi: 10.1021/acsami.5c25104 (PMC13133783; doi:10.1021/acsami.5c25104)
Supplement: Supplementary file 1 [file am5c25104_si_001.pdf]

Supplemental Information for Oxygen Vacancy Evolution at $Li_xV_2O_5$/LiPON Solid State Electrochemical Interfaces Using Depth Resolved Cathodoluminescence Spectroscopy

Daniel Halbing[1], Gregory Pustorino[2], Leopoldo Tapia-Aracayo[3], David Stewart[3], Yue Qi[2], Leonard J. Brillson*[1,4]

[1]Department of Physics, The Ohio State University, Columbus, OH 43210, USA

[2]Department of Engineering, Brown University, Providence, RI 02912, USA

[3]Department of Materials Science and Engineering and Institute for Research in Electronics and Applied Physics, University of Maryland, College Park, Maryland 20742 USA

[4]Department of Electrical and Computer Engineering, The Ohio State University, Columbus, Ohio, 43210 USA

*: corresponding author: brillson.1@osu.edu

Table of Contents

I. DRCLS Monte Carlo Simulation of V_2O_5/LiPON……………………………………2

II. Electrochemical Impedance Spectroscopy of V2O5/LiPON…..……………………..3

III. Depth Profile of V_o 1.6 eV Peak in Standard Annealing Vs. in Oxygen Annealing…..4

IV. DRCLS Comparison Between Pure V_2O_5 Phases and Cycled V_2O_5………………….5

I. DRCLS Monte Carlo Simulation of V_2O_5/LiPON

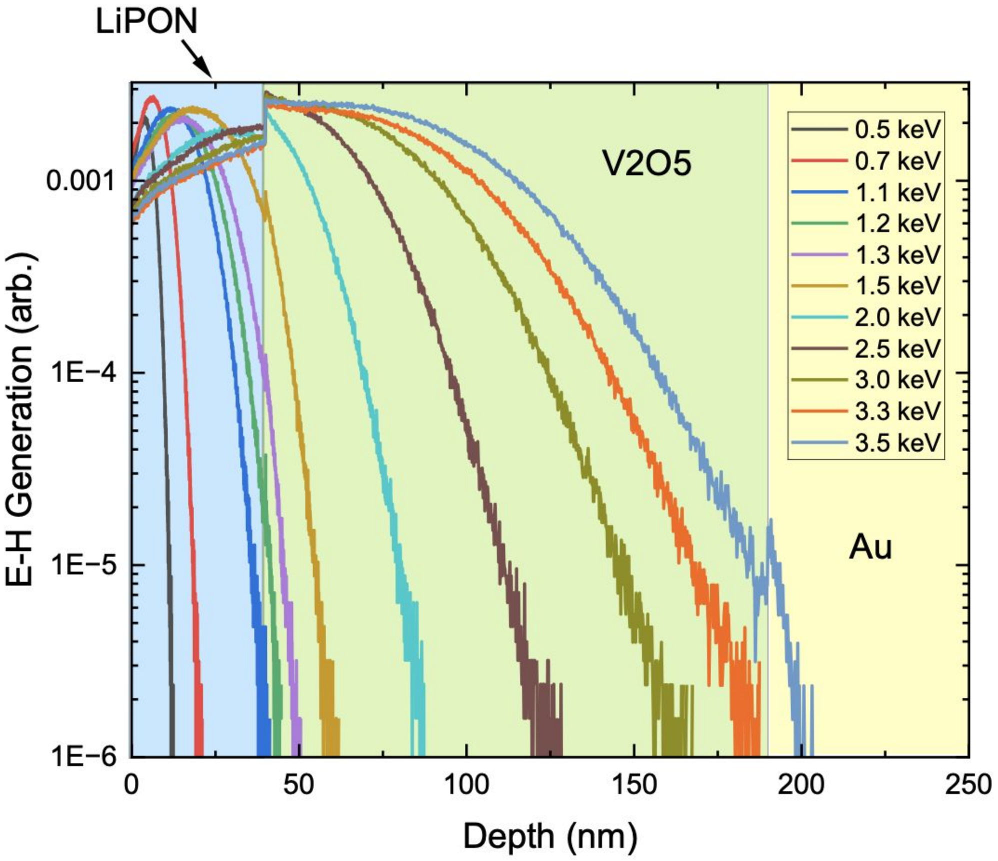

Figure S1. Monte Carlo simulation of V_2O_5/LiPON sample stack structure. The blue region indicates the LiPON layer, while the green region indicates the V_2O_5 layer, with the yellow region being the bottom gold contact. Each colored curve represents a different electron gun beam energy.

Figure S1 shows the penetration depths for each respective beam energy obtained through Monte Carlo simulations done on the Casino 2.5.1.0 software for the samples investigated in this study. For this simulation, 2,000,000 incident electrons were simulated per beam energy with a specimen tilt angle of 44.7 degrees at beam energies of 0.7 keV, 1.1 keV, 1.2 keV, 1.3 keV, 1.5 keV, 2.0 keV, 2.5 keV 3.0 keV, 3.3 keV, and 3.5 keV. The specimen structure consisted of a 40 nm top layer of LiPON, followed by 150 nm of V_2O_5, with a bottom gold contact. Higher beam energies penetrate deeper into the sample, with the interfacial region being penetrated at ~1.2 keV.

II. Electrochemical Impedance Spectroscopy of V_2O_5/LiPON

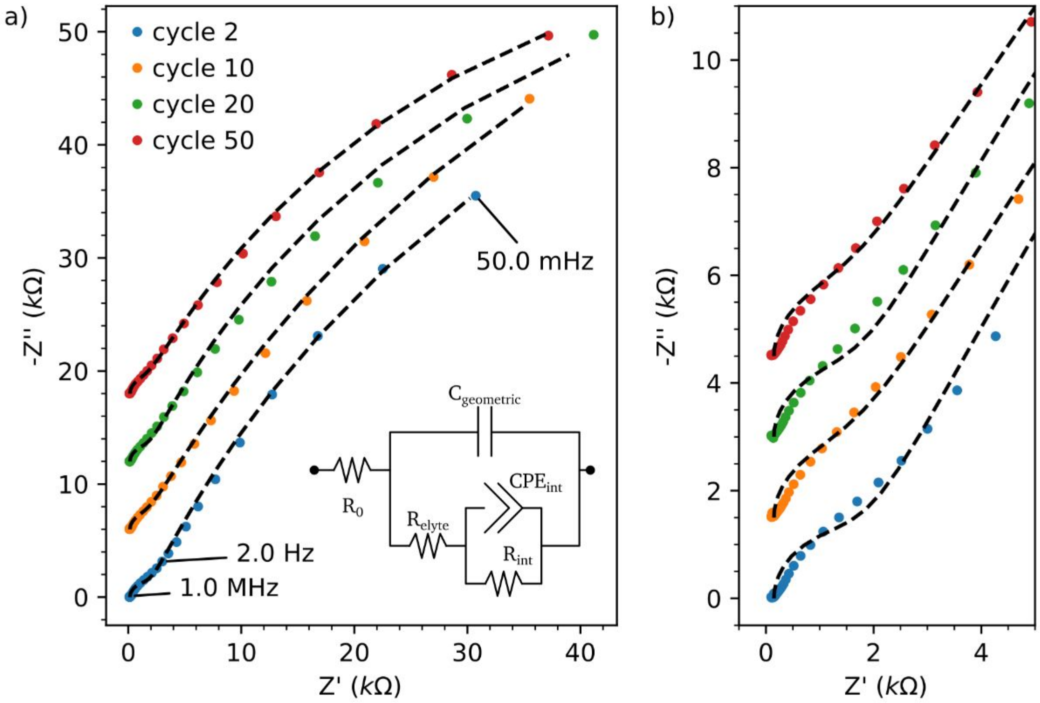

Figure S2. (a) Nyquist plot of electrochemical impedance spectroscopy (EIS) data measured at 4 V on a device over 50 cycles, with dashed lines showing the fitted model. Inset is the equivalent circuit used to model the EIS data and derive the interface properties shown in Figure 2 of the main text. (b) Same as (a) with axes scaled to show the high frequency region.

EIS was measured between 1 MHz and 50 mHz at 6 points per decade and 2 measurements per point using an amplitude of 20 mV and a wait time between measurements of 10% of the period. A simplified equivalent circuit model was developed to reduce the number of free variables and improve the reliability of the fitting. Separate measurements of the LiPON and V_2O_5 thin films between Au gave fixed resistance values of 2000 and 5 Ω, respectively. R_0 represents the contact resistance and electronic resistance of Li foil, fixed at 150 Ω. R_{elyte} represents the ionic resistance of the 1 M $LiClO_4$ and LiPON electrolytes, and is dominated by the resistance of the LiPON. The geometric capacitance of the cell is left to be fit.

The LiPON/V_2O_5 interface is modeled using a constant phase element (CPE_{int}) for the accumulation of charge at the interface in parallel with a resistance R_{int}, representing the faradaic transfer of Li^+ across the interface. The charge transfer resistance is most strongly related to the kinetics of the Li redox reaction. The accumulation of net charge resulting in an interface capacitance may be due to Li^+, e^-, and unbalanced oxygen vacancies. Since the EIS was conducted in a delithiated state and away from potentials for electrochemical reactions, the participation of Li^+ in the V_2O_5 is expected to be minimal.

III. Depth Profile of V_o 1.6 eV Peak in Standard Annealing Vs. in Oxygen Annealing

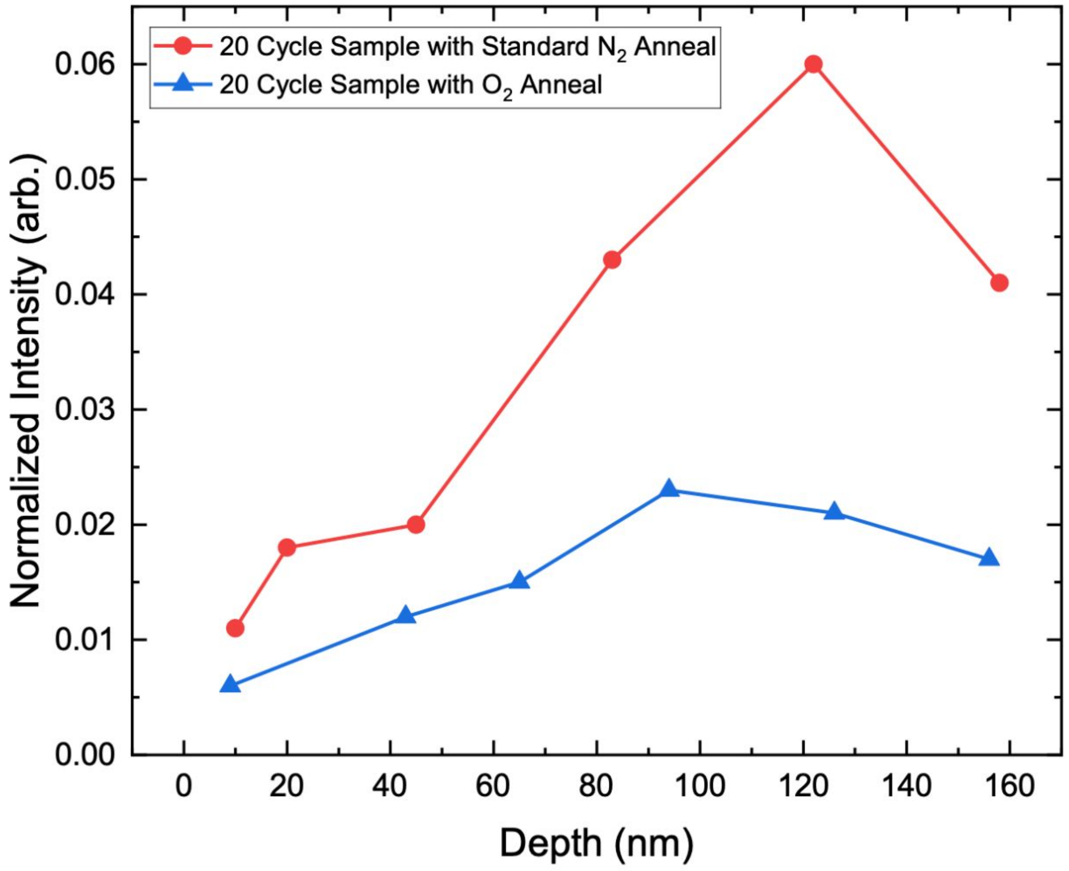

Figure S3. Depth profile plot of V_o 1.6 eV defect peak in one 20-cycle sample that underwent standard annealing (N_2) annealing and one 20-cycle sample that underwent O_2 annealing. The sample that underwent O_2 annealing had significantly lower intensity of oxygen vacancies throughout, confirming the theoretical results that the 1.6 eV defect peak is in fact linked to oxygen vacancies.

IV. DRCLS Comparison Between Pure V_2O_5 Phases and Cycled V_2O_5

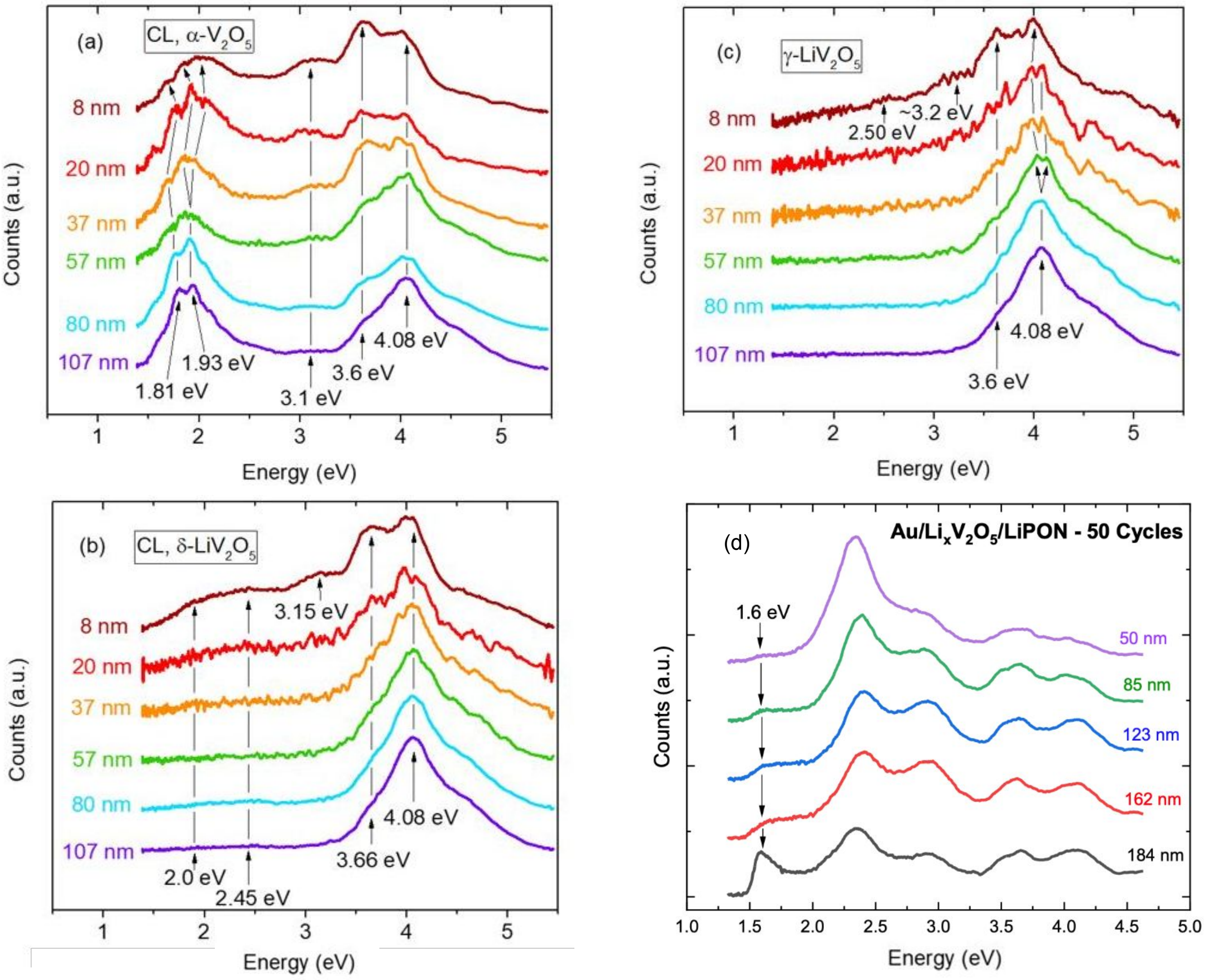

Figure S4. DRCLS comparison of pure (pristine) V_2O_5 phases with cycled $Li_xV_2O_5$. There are three pristine phases of V_2O_5: (a) α-V_2O_5, (b) δ-V_2O_5, and (c) γ-V_2O_5. The DRCLS spectra of cycled $Li_xV_2O_5$ are shown in (d). Peaks with energy ~3.6 eV and ~4.0 eV are common to all phases; however, the 3.6 eV peak has the highest intensity at most depths for α-V_2O_5 while ~4.0 eV dominates at most depths for δ-V_2O_5 and γ-V_2O_5. The remaining peaks are unique to each individual phase and therefore can be used as phase identifiers. Comparing the pure phases (a, b, and c) to the cycled spectra (d), the ~3.6 eV and ~4.0 eV peak intensities remain similar relative to each other throughout, which likely indicates a mixing of phases is present as neither peak dominates. We also see peak energies of ~2.0, 2.45, and 3.2 eV, which are similar to different characteristic peaks from all of the pure phases. This further indicates that there is a mixture of phases in the cycled $Li_xV_2O_5$. A mixture of phases therefore also means we have a mixture in valence states for the vanadium ions, as we expect we expect 5+ for V in α-V_2O_5, expect both 5+ and 4+ for V in δ-V2O5, and expect primarily 4+ for γ-V_2O_5. Reprinted with permission of Journal of Materials Chemistry A. [12]
